# Supplementary material for: A Critical View of the Application of the APEX Software (Aqueous Photochemistry of Environmentally-Occurring Xenobiotics) to Predict Photoreaction Kinetics in Surface Freshwaters
Source: Molecules. 2019 Dec 18;25(1):9. doi: 10.3390/molecules25010009 (PMC7017383; doi:10.3390/molecules25010009)
Supplement: Supplementary file 1 [file molecules-25-00009-s001.zip › 3.2.4_gcc-4.4.0.zip!/3.2.4_gcc-4.4.0/3.2.4_gcc-4.4.0/doc/octave/html/interpreter/Image-Processing.html]

Image Processing - Untitled


Next: Audio Processing,
Previous: Signal Processing,
Up: Top


---

## 31 Image Processing

Since an image basically is a matrix Octave is a very powerful
environment for processing and analyzing images. To illustrate
how easy it is to do image processing in Octave, the following
example will load an image, smooth it by a 5-by-5 averaging filter,
and compute the gradient of the smoothed image.

```
     I = imread ("myimage.jpg");
     S = conv2 (I, ones (5, 5) / 25, "same");
     [Dx, Dy] = gradient (S);
```

In this example `S` contains the smoothed image, and `Dx`
and `Dy` contains the partial spatial derivatives of the image.

- Loading and Saving Images- Displaying Images- Representing Images- Plotting on top of Images- Color Conversion
